# Phenotypic Plasticity Promotes Overwintering Survival in A Globally Invasive Crop Pest, *Drosophila suzukii*

**DOI:** 10.3390/insects9030105

**Published:** 2018-08-21

**Authors:** Dara G. Stockton, Anna K. Wallingford, Gregory M. Loeb

**Affiliations:** 1 Department of Entomology, Cornell AgriTech and New York State Agricultural Experiment Station, Cornell University, Geneva, NY 14456, USA; gme1@cornell.edu; 2 Invasive Insect Biocontrol and Behavior Laboratory, USDA–ARS, Beltsville, MD 20705, USA; annawllngfrd@gmail.com

**Keywords:** spotted wing drosophila, SWD, cold hardening, acclimation, small fruit, physiology, winter morph

## Abstract

Spotted wing drosophila, *Drosophila suzukii* Matsumura, is a major pest of small fruit worldwide in temperate and subtropical growing regions. In Northern climates, *D. suzukii* likely overwinters locally under leaf litter and snow pack, but our understanding of the factors affecting thermal susceptibility is limited. While previous investigations of thermal susceptibility in this species have employed conventional static acclimation protocols, we aimed to determine whether gradual cooling, or dynamic acclimation, may extend the limits of known thermal tolerance by more closely approximating naturally occurring shifts in temperature. First, we assessed survival among adult and pupal *D. suzukii* using static acclimation. Then, we re-assessed survival using a novel dynamic acclimation method. We found that while static acclimation was sufficient to induce cold tolerance, dynamic acclimation significantly improved survival at temperatures as low as −7.5 °C. Following static acclimation, the lower lethal limit of adult *D. suzukii* was −1.1 °C in winter morphotype (WM) adults compared to 1.7 °C in non-acclimated summer morphotype (SM) adults. Dynamic acclimation reduced the lower limit to −5 °C in SM flies. At the end of our study 50% of WM flies survived 72 h at −7.5 °C. Below 0 °C pupal survival declined significantly regardless of acclimation procedure. However, pupal acclimation improved survival outcomes significantly compared to non-acclimated pupae, suggesting that while juvenile diapause is unlikely, cold hardening likely benefits those flies which may develop into the overwintering WM population. These data suggest that the degree of cold hardening is proportional to the thermal environment, a finding previously unrecognized in this species. Given the economic impact of this pest, these data may have important implications for offseason population monitoring and management. We discuss how phenotypic plasticity may drive geographical range expansion, and the impact of climate change on the spread of this species.

## 1. Introduction

Spotted wing drosophila, *Drosophila suzukii* Matsumura (Diptera: Drosophilidae), is a major invasive pest of small fruit crops in North America, South America, and Europe [1,2,3,4], with potential to spread to Africa and Oceania [4]. Unique among its sister Drosophilids in the melanogaster group, this species, which originated in Northeast Asia, possesses a serrated ovipositor used for oviposition into ripening and unripe fruit [5,6,7,8]. As such, *D. suzukii* is responsible for significant economic losses in small fruit crops worldwide, including raspberries, blueberries, strawberries, and cherries [9,10]. In Northern climates, *D. suzukii* reaches peak populations in the summer, which coincides with warm temperatures and peak fruit abundance [5,11,12,13]. Subsequently, populations decline in the winter, and detection is often difficult until temperatures increase again in the spring. Winter trapping studies in Europe and North America have captured *D. suzukii* despite periods below 0 °C [11,14,15], suggesting that *D. suzukii* may overwinter locally, similar to *D. melanogaster* [16,17,18,19]. The time of first capture varies yearly and regionally, and is presumably affected by the degree and duration of extreme cold temperatures to which the insects were exposed the previous winter. However, the behavioral and physiological mechanisms by which this relatively cold-intolerant insect [20,21,22] survives harsh winters remains unclear. 

At both high and low temperatures, physiological functions in *D. suzukii*, including larval development and reproduction, become dysregulated [14,23,24,25,26]. However, phenotypic plasticity and the development of certain traits in response to winter weather conditions appears to be an important thermoregulatory survival strategy common among many temperate Drosophilids [27,28,29]. Whereas summer-morphotype (SM) flies develop under conditions of long day length and warm temperatures, winter-morphotype (WM) traits, including longer wing length, larger bodies, and darker cuticular pigmentation, occur when reared under cool conditions (10–15 °C) [5,11,22,30,31,32,33,34]. The traits associated with WM flies appear adaptive and allow the insect to absorb and retain heat throughout the winter [35,36,37,38], although reduced water loss and improved immune function have also been documented [34]. 

WM flies consistently display improved recovery and longevity at cold temperatures compared to SM flies [31,32,33]. Indeed, bioinformatics data indicates changes in ion transport and carbohydrate metabolism among WM Drosophilids [30,32,34,39], consistent with physiological cold tolerance and the prevention of ice crystal formation within the haemocoel [40,41]. However, this species appears to be relatively freeze intolerant [20,31,42], and appears to employ a freeze-avoidant strategy similar to most other Drosophilids [43]. One study observed cold shock survival at temperatures below 0 °C for up to 1 h and found −7.5 °C to be the lower lethal limit [20]. Another identified supercooling points near −20 °C, although the exposure period at these temperatures was approximately 1 second [31]. In contrast, freeze tolerant species are known to survive temperatures as low as −40 °C for extended durations [44].

There are currently no known reports of juvenile overwintering by larval or pupal *D. suzukii* and juvenile overwintering is generally uncommon among temperate Drosophilids [37]. Many reports suggest lesser tolerance among larvae and pupae compared to adults, and little survival at temperatures below 5 °C [22,31,37,45,46]. One recent study reported an Lt_50_ of 21 h for pupae at 5 °C [46]. However, the pupae were not acclimated, and it is unclear how developmental acclimation may affect their survival. There are select temperate Drosophilids which do overwinter in juvenile form, including *Chymomyza costata* Zetterstedt, a holarctic species which is found in the Northern United States, Canada, and Europe [47], and is known to undergo functional larval diapause [43,44,48]. Interestingly, gradual cooling appears to play an important role in the freeze tolerant response of *C. costata* [49].

Previous investigations into the thermal susceptibility of *D. suzukii* have used conventional static acclimation protocols, in which the insect is kept at a single constant cool temperature, typically 10–15 °C, for the duration of the acclimation period prior to testing [20,22,31,32,33,50]. To our knowledge, there is no research on gradual cold hardening, or dynamic acclimation, in *D. suzukii*; and the effect this may have on our estimations of thermal susceptibility—whether acclimatization at temperatures below 10 °C would extend the known thermal tolerance limits identified in this species—remains unknown. The plasticity of thermal thresholds among arthropods is certainly documented [51,52], particularly among those found near the poles [53]. Exposure to extreme temperatures during a critical acclimation period is often sufficient to adaptively [52] shift chill coma and coordination thresholds, as well as to reduce the lower lethal limit of the organism [53]. In *D. suzukii*, although 10–15 °C is sufficient to induce WM traits, the physiological changes associated with extreme cold hardening (i.e., freeze tolerance) may require longer durations at cooler temperatures than has been explored. This is significant because in nature, cooling tends to occur incrementally over time, providing opportunity to make physiological and behavior modifications potentially important for survival [54,55].

Our aim in this study was to further the discussion of overwintering potential in *D. suzukii* and revise our current estimations of thermal susceptibility in *D. suzukii* with consideration for dynamic acclimation and physiological plasticity. Broadly, we compared survival following static vs dynamic acclimation in response to short and long-term cold exposure durations. We also aimed to re-evaluate the overwintering potential of juvenile *D. suzukii*, addressing acclimation effects within the context of survival and development. Because the degree of cold hardening is theoretically proportional to experience with cool temperatures [21,56,57,58], we hypothesized that a gradual reduction in temperature would enhance cold hardening and possibly confer freeze tolerance. Our dynamic acclimation method is a novel approach to studying thermal susceptibility in *D. suzukii* and may provide a more accurate estimate of overwintering potential of *D. suzukii* than previous reports. Given the wide geographic distribution of *D. suzukii*, and the threat of range expansion due to climate change [59,60,61,62], it may be increasingly important to understand physiological plasticity in order to predict the long-term expansion potential [50,63] of this species.

## 2. Materials and Methods

### 2.1. Insect Colony

For the laboratory assays described here, the SM *D. suzukii* colony was maintained at the Cornell AgriTech facility in Geneva, NY. The colony was established from wild caught flies captured on farms near Geneva, New York. The original colony began in 2011, and in 2014, 2016, and 2017, additional wild flies were added to the colony to help maintain genetic diversity. The captures occurred during the summer months of June–September, when wild populations were highest. The colony was maintained on a standard cornmeal diet [64] in 8 oz. polypropylene fly rearing bottles (VWR^®^ International; Radnor, PA, USA). The diet was replaced weekly to ensure colony health. The colony was maintained in a climate-controlled growth chamber set to 25 °C, with a 14 L: 10 D photoperiod, and 65% relative humidity (r.h.) to simulate “summer conditions” [33].

### 2.2. Static Susceptibility in Adults

First we investigated the extent to which static acclimation may confer protection against chronic cold exposure in adults. We directly compared cold tolerances in the three most commonly reported phenotypes associated with conventional static acclimation studies: (1) developmentally acclimated WM flies, (2) SM flies acclimated as adults, and (3) non-acclimated SM flies. This allowed us to measure thermal susceptibility in a manner similar to that previously reported [20,22,31,32,33].

**Figure 1:**
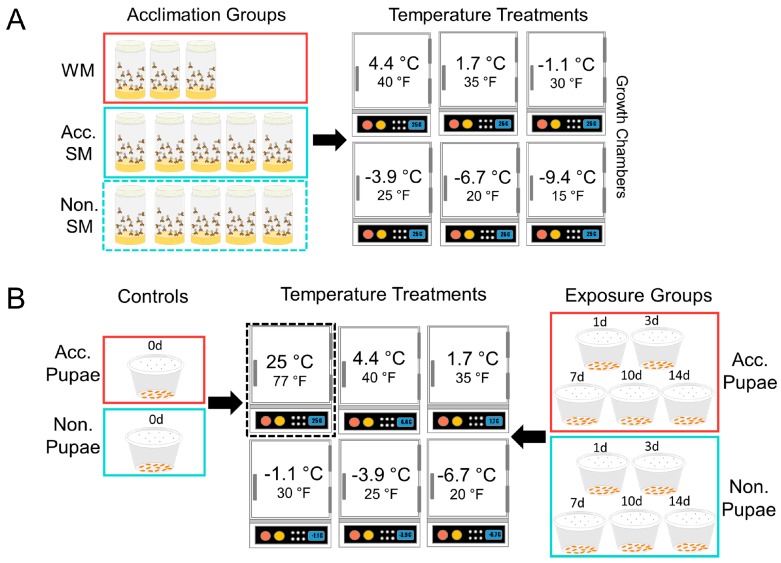
Experimental design of the adult (**A**) and pupal (**B**) static susceptibility tests. Group labels: “WM” refers to developmentally acclimated WM flies, “Acc. SM” refers to acclimated SM flies, and “Non. SM” refers to non-acclimated SM flies.

**Figure 2:**
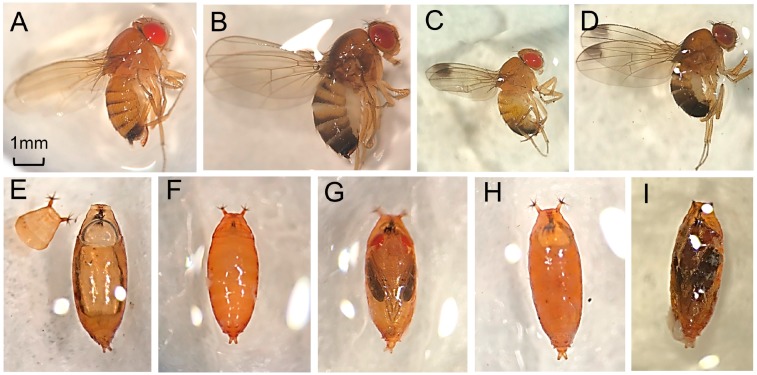
Differences in *D. suzukii* phenotypic expression of size and pigmentation when reared at warm (**A**,**C**) and cool (**B**,**D**) temperatures. Adult SM female (**A**), adult WM female (**B**), adult SM male (**C**), and adult WM male (**D**). Cast pupal case from a recently eclosed *D. suzukii* (**E**), healthy undeveloped pupa 2 days post-pupation (**F**), healthy developed pupa 5 days post-pupation (**G**), dead undeveloped pupa (**H**), dead developed pupa (**I**).

We used a within-subject experimental design and exposed adults to six different temperatures (4.4, 1.7, −1.1, −3.9, −6.7, −9.4 °C) (40, 35, 30, 25, 20, 15 °F) for up to 4 weeks (Figure 1A). For a given temperature treatment, the same flies were monitored at each time point. We included 3 acclimation treatments, non-acclimated SM flies (Figure 2A,C), acclimated SM flies, and developmentally acclimated WM flies (Figure 2B,D), crossed with 6 temperature levels resulting in a 3 × 6 factorial design. We monitored three experimental replicates among WM flies, and 5 replicates among acclimated and non-acclimated SM flies for each temperature tested (Table 1). There were approximately 25 mixed-sex flies per replicate, contained in a single fly bottle, but the total number of insects in each replicate varied slightly. The fly sex ratios were approximately equivalent in each bottle. The replicate bottles contained 50 ml cornmeal diet as a source of nutrition, which was replaced weekly.

We reared WM flies by transferring eggs to a 15 °C incubator for the duration of development [33]. The eggs were transferred in the colony bottles in which they were laid, in a cornmeal diet agar. Development time at this temperature increased from approximately 13 days at 25 °C to approximately 28 days. Adult WM flies were aged 5–7 days at 15 °C prior to use. We induced cold-acclimated SM flies by moving newly eclosed adults to the 15 °C incubator for 5 days. Acclimation cohorts were kept in groups of 25 flies. Temperature and humidity were regulated in six individual environmental growth chambers (model I-30BL: Percival Scientific, Inc.; Perry, IA, USA), which were monitored daily with HOBO^®^ data loggers (model UX100-011 Temp/RH; Onset Computer Corporation, Bourne, MA, USA) to ensure the settings were accurate. The growth chambers were kept on a 10 L: 14D light cycle to simulate winter photoperiodic conditions. Humidity decreased to 25% r.h. to limit ice formation.

The flies were monitored for mortality at set intervals at 1, 3, 7, 10, 14, 21, and 35 days. Mortality assessments required a period of revival, particularly at the lower temperature treatments [56,65]. For this reason, mortality was assessed after a warm-up period. We moved the flies from their respective cold chambers into the colony room (25 °C) for two hours prior to assessment [29].

### 2.3. Pupal Susceptibility

Next, we assessed the effect of temperature on pupal survival and observed both survival and development at above and below 0 °C for up to two weeks and compared our results among cold-acclimated and non-acclimated pupae. We used a between-subjects experimental design and exposed pupae to cold temperatures at five temperatures (4.4, 1.67, −1.11, −3.87, −6.7 °C) and for five exposure durations (1, 3, 7, 10, 14 days; Figure 1B). We compared survival at these temperatures among cold-acclimated and non-acclimated pupae. As a result, we employed a 2 × 5 × 5 factorial design with two acclimation treatments, 5 temperature treatments, and 5 exposure duration treatments, for a total of 50 treatment groups with *N* = 25 pupae per treatment group. Because mortality assessments required that we rear the pupae out to adults, we compared survival between groups at each individual time point. Each group was independent of the other treatments.

Cold-acclimated pupae were induced by moving eggs and 1st instar larvae into a 15 °C chamber for the duration of their development [37]. The light cycle in the 15 °C chamber was set at 10 L: 14 D with 60% r.h. Flies in the cold acclimation and non-acclimation treatments were matched for age so that pupation in both groups occurred within 1–2 days of each other. After pupation, 1–2 days old pupae from both acclimation treatments were removed from the agar in the rearing bottles and cleaned. We cleaned the pupae by carefully extracting them from the cornmeal agar of their rearing bottles using a long metal spatula. Cleaning the pupae increased our transfer survival rates (from 50% to close to 100%). We rinsed the pupae with distilled water to clear the respiratory horns, and surface sterilized them with a rapid 95% acetone and 95% ethanol rinse, to remove any surface pathogens that could affect survival throughout the experiment. This was followed by an additional rinse with distilled water to remove any remaining acetone or ethanol on the insect cuticle. The pupae were then transferred to a paper towel to absorb excess moisture before being moved to their final destination. 

The cleaned pupae were moved in groups of 25 into 95% ethanol-sterilized, 16 oz. transparent polypropylene deli cups (473 ml, 11.7 × 7.6 × 8.9 cm; Pro-Kal™ PK165-C; Fabri-Kal, Kalamazoo, MI, USA). Each cup contained a complete treatment group. All treatment groups were independent. The lid of the deli cup was perforated 10× with a small dissection probe (product No. 4751; BioQuip Products, Rancho Dominguez, CA, USA) to allow ventilation. After set-up, the pupae were immediately transferred to their respective cold growth chambers. The growth chambers were the same as those used in the adult susceptibility experiment and were kept at the same photoperiod and humidity settings.

When the designated thermal exposure time had lapsed, the pupae in their deli cups were removed from the growth chambers. Two groups, a cold-acclimated and a non-acclimated group, were removed from each temperature chamber at each time point. As a result, each time point, 10 total treatment groups were removed and transferred to an observation room, held at 25 C, 60% r.h., 14:10 L:D. We then observed eclosion (Figure 2E) and pupal development for up to 7 days. We scored eclosion, including partial eclosion, as evidence of survival. Failure to eclose after 7 days indicated that the pupae had died at some point during the experiment. As a control, we also compared survival among acclimated and non-acclimated pupae without additional chill time, which were moved directly to the warm colony chamber at 25 °C to complete development. This allowed us to assess baseline survival.

To differentiate between those pupae that continued to develop throughout the experiment, and thus survived past the point of direct thermal exposure (the point at which we moved the pupae from the cold chamber to the warm-up room), we observed the pupal development stage in all dead pupae at the end of the experiment. We marked those pupae as either undeveloped or developed. Undeveloped pupae (Figure 2F,H) were characterized by internal white or brown coloration without clear differentiation of adult tissues visible through the pupal case. Developed pupae (Figure 2G,I) had red, clearly defined eye spots and black striping along the mid-point of the pupal case indicating wing formation.

### 2.4. Dynamic Acclimation

Finally, we observed the effect of dynamic acclimation on thermal susceptibility in adult *D. suzukii* and pupae. We slowly decreased the temperature from 15 °C to −7.5 °C over the course of 3 weeks, using three-day intervals for temperature reduction, and observed the survival of SM and WM adults and pupae. The temperature settings were monitored closely and adjusted daily as needed. We moved SM and WM flies into bottles in groups of approximately 15–20 flies. Each bottle represented an experimental replicate. There were 10 WM fly bottles (*N* = 147 total flies), and 10 SM fly bottles (*N* = 196). Three additional bottles of WM (*N* = 43) and SM flies (*N* = 67) were treated as controls and were kept in the 15 °C chamber for the duration of the experiment. This allowed us to control for the effects of time in our study. These treatments are denoted as C-SM and C-WM to indicate their status as control groups. Additionally, at each temperature below 0 °C, we moved three new bottles of SM (*N* = 47) and WM flies (*N* = 38) into the cold chamber, designated as “acute SM” and “acute WM”, respectively. This allowed us to directly compare survival among flies in the gradual cooling treatment and flies similar to that used in our static susceptibility tests.

Survival was assessed daily by visual examination. Because chill coma occurred in the majority of flies below 0 °C, particularly SM flies, a revival period was required at our lower temperatures in order to assess mortality. Below 0 °C, the bottles were removed from the cold chamber daily and given 1–2 h to warm-up at 25 °C prior to assessment. As chill coma recovery times increased with decreasing temperature, revival times were longest at our coldest temperatures.

Activity level was also recorded to determine how acclimation affects movement in *D. suzukii*. We used a 3-point scale and recorded the overall activity level of each treatment group daily, with three being the most activity, and 0 being the least. An activity score of three was assigned if the insects were actively flying within the bottles when they were removed from the cold chamber. A score of 2 indicated that they were walking, but not actively flying. A score of 1 indicated that they were standing, but not moving around the bottle. If touched with spatula, flies with a score of 1 would move aside. A score of 0 indicated chill coma. If touched with a spatula, the flies would fall over and remain unresponsive until they were revived after a period of time at 25 °C.

We measured pupal susceptibility in this experiment as well. Bottles of < 1 day old non-acclimated pupae were held in the same cold chamber as the adults. After three days at each temperature, two bottles were removed and held at 25 °C for 7 days. Pupal survival was assessed using the same method previously described - we recorded the number of empty pupal cases, indicative of eclosion, the number of pupae that failed to eclose but displayed features consistent with advanced development, and the number of undeveloped pupae. 

In all treatment bottles, accumulated condensation along the bottle walls was removed daily with a sterile wipe to prevent drowning. Throughout the course of the experiment, the flies were kept in rearing bottles (described previously), with 50 ml of standard cornmeal-agar diet. The bottles were replaced once weekly, or as needed if the diet became sticky or wet with condensation.

### 2.5. Statistical Analysis

Adult survival was assessed with generalizing estimating equations (GEE) modeling as the data were not independent at each time point [66]. GEE analysis was performed with the package *geepack*, using a binomial distribution (family = binomial) and an independent correlation structure (corstr = “independence”). The scale parameter was fixed at 1, rather than estimated, because the data were binary (scale.fix = T). We used a generalized linear model (GLM) with binomial distribution to assess the effects of temperature, duration of exposure, and acclimation treatment on survival and development ratios of pupal *D. suzukii, followed by analysis of deviance*.

We used GEE modeling for analysis of the dynamic acclimation data and determined the effects of treatment, temperature, and time on survival. Differences in survival between our four treatments: SM, C-SM, WM, and C-WM were compared with general linear hypothesis testing (GLHT) for posthoc multiple comparisons with Tukey contrasts, using the package *multcomp*. A separate GLM isolating treatment as a factor was used as the base model for our posthoc analyses.

A simple linear regression was used to predict activity level based on temperature. We compared activity level among our treatment groups with t-tests. Pupal survival and development following dynamic acclimation was assessed using a GLM with binomial distribution, followed by analysis of deviance and pairwise chi-squared tests for multiple comparisons of pupal survival. Analyses were performed in R i386 (version 3.4.0; the R Foundation for statistical computing (platform x86_64-w64-mingw32/x64); Vienna, Austria).

## 3. Results

### 3.1. Adult Susceptibility

**Figure 3:**
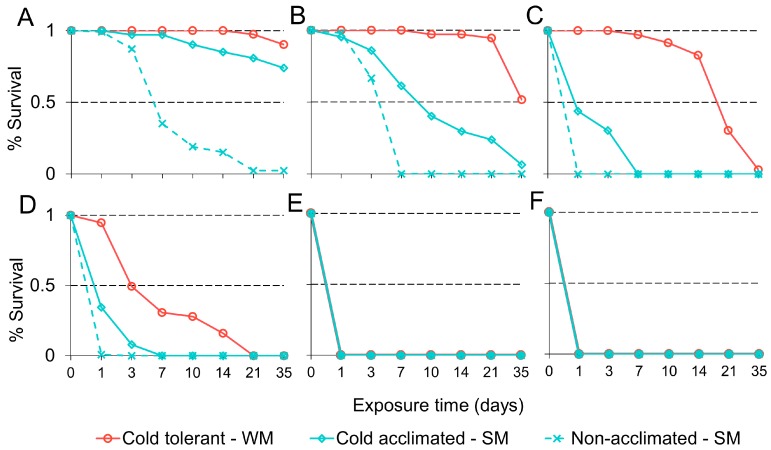
Mean percent survival (y-axis) of adult *D. suzukii* with varying states of cold hardiness in response to 4.4 (**A**), 1.67 (**B**), −1.11 (**C**), −3.87 (**D**), −6.67 (**E**), or −9.4 °C (**F**) chronic cold exposure for up to 35 days (x-axis). Line color indicates cold-hardiness treatment group: developmentally acclimated WM flies (solid red line), cold-acclimated SM flies (solid blue line), and non-acclimated SM flies (dashed blue line).

We found that long-term developmental exposure to cool temperatures (WM flies) significantly improved survival in adult *D. suzukii* in response to acute and sustained cold exposure, compared to cold-acclimated and non-acclimated SM adults (χ^2^ = 64.4, df = 2, *p* < 0.001; Figure 3A–F). Temperature was a significant factor affecting survival (χ^2^ = 241.5, df = 1, *p* < 0.001), as well as the duration of exposure (χ^2^ = 115.2, df = 1, *p* < 0.001). At 4.4 °C there was no difference between cold tolerant and cold-acclimated adults. However, at 1.67 °C and below, there was a decline in survival compared to cold tolerant WM adults.

### 3.2. Pupal Susceptibility

**Figure 4:**
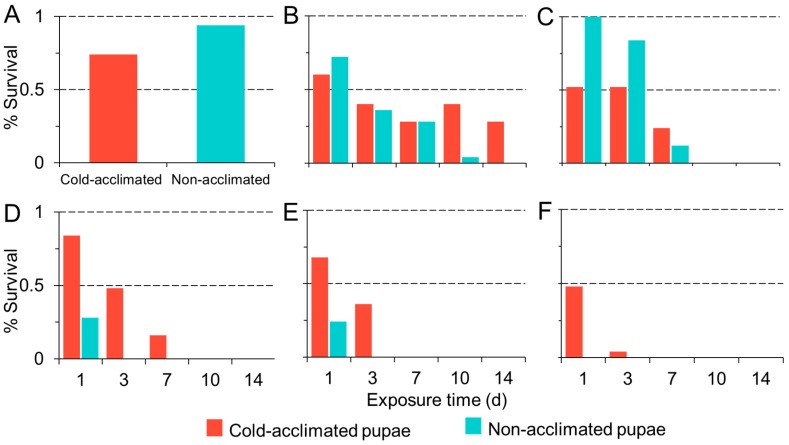
Percent pupal survival (y-axis) of cold-acclimated (blue bars) and non-acclimated (red bars) *D. suzukii* held at 25 (**A**), 4.4 (**B**), 1.87 (**C**), −1.11 (**D**), −3.87 (**E**), and −6.67 °C (**F**) for up to two weeks (x-axis).

The baseline survival of our control flies (flies not subjected to a chill schedule) in the cold-acclimated and non-acclimated treatment groups was statistically different, with greater survival among non-acclimated pupae (χ^2^ = 7.44, df = 1, *p* = 0.006; Figure 4A). Among flies in our chill treatment groups, pupal survival was temperature dependent and decreased significantly with decreasing temperature (χ^2^ = 256.46, df = 1, 52, *p* < 0.001; Figure 4B–F). Survival decreased with increasing duration of exposure (χ^2^ = 237.8, df = 1, 50, *p* < 0.001). Overall, acclimation had a significant effect on survival, with greater survival among cold-acclimated pupae than non- acclimated pupae (χ^2^ = 29.4, df = 1, 51, *p* < 0.001). No flies in the non-acclimated treatment survived below 0 °C after 24 h exposure. In contrast, we observed 4% survival among acclimated pupae after 72 h exposure to −6.7 °C.

**Figure 5:**
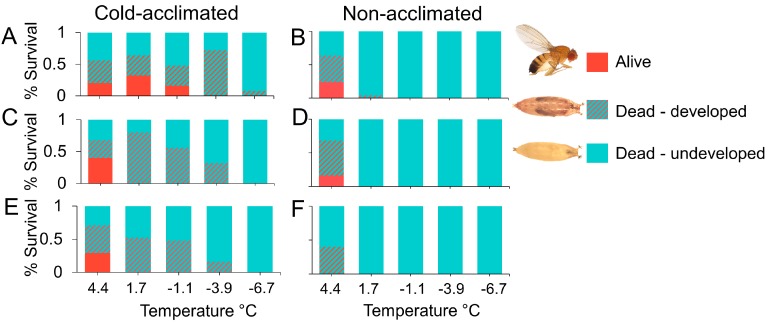
Percent survival and development of cold-acclimated (**A**, **C**, **E**) and non-acclimated (**B**, **D**, **F**) pupae exposed to low temperatures for 7 days (**A**-**B**), 10 days (**C**-**D**), or 14 days (**E**-**F**). Red bars indicate insects that eclosed during the observation period.

We also observed a difference in development among acclimated and unacclimated pupae (Figure 5A–F). In addition to greater survival among acclimated pupae (Figure 5A,C,E), we also observed more complete development compared to unacclimated pupae (Figure 5B,D,F) (χ^2^ = 90.68, df = 1, 28, *p* < 0.001). Unacclimated pupae were more likely to lack late-stage developmental features such as wings, eyes, and legs, which are visible through the pupal casing. In acclimated flies we observed developed pupae at all temperatures at 7 days. At 10 days and 14 days, pupal development continued up to −3.9 °C. In non-acclimated flies, comparatively, pupal development appeared to arrest below 4.4 °C. 

### 3.3. Dynamic Acclimation

**Figure 6:**
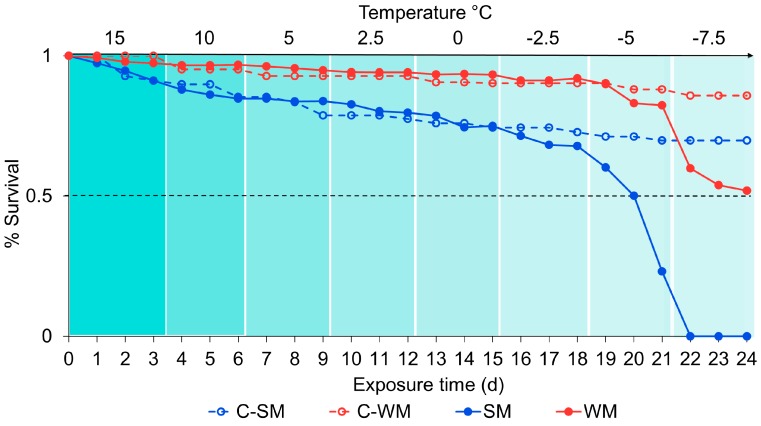
The effect of gradual cooling on mean survivorship in adult SM and WM *D. suzukii*. Survivorship was observed (y-axis) as the temperature (x-axis TOP) decreased over time (x-axis BOTTOM) in 3 days intervals. Color bands indicate differences in temperature, with lighter bands being cooler than darker bands. The solid dots and lines indicate percent survival per replicate among flies in the gradual cooling treatments (SM and WM). Open dots and dashed lines indicate flies in the control treatments (C-SM and C-WM) which were kept at 15 °C for the duration of the study.

There was a significant effect of treatment on survival outcomes (χ^2^ = 193, df = 3, *p* < 0.001; Figure 6). WM flies survived in higher proportions than SM flies in both the experimental condition (gradual cooling) (z = 21.66, *p* < 0.001) and the control (z = 8.56, *p* < 0.001). Above −5 °C, WM flies survived as well as C-WM flies, which were held at 15 °C for the duration of the study (z = 0.99, 0 = 0.75). Similarly, SM flies survived as well as C-SM flies above −5 °C (z = −0.19, *p* = 1.00). 

Temperature was also a significant factor (χ^2^ = 312, df = 1, *p* < 0.001). Survival remained high above −5 °C. After 72 h at −5 °C, the proportion of surviving SM flies declined from approximately 68% to 23%. In contrast, there was only a 7% decline in survival among WM flies during this period. At −7.5 °C no SM flies survived 24 h exposure. However, after 72 h exposure to −7.5 °C, approximately 52% WM flies survived. Of the surviving WM flies at the end of the experiment, 33% were male and 67% were female. 

Although time was a significant factor predicting survival outcomes (χ^2^ = 155, df = 1, *p* < 0.001), we decreased temperature over time making this is difficult to interpret. We did not observe a large decrease in survival over time among our control groups (C-SM and C-WM flies), which were not gradually cooled, but kept at 15 °C for the duration of the study to monitor baseline survival over time as the flies aged. Among C-SM flies, survival was approximately 70% after 24 days (the end of the study), and 86% among C-WM flies. Survival in these groups gradually declined from the start of the experiment and survival among the experimental groups was similar to the controls up until day 19, when the temperature was reduced to −5 °C.

**Figure 7:**
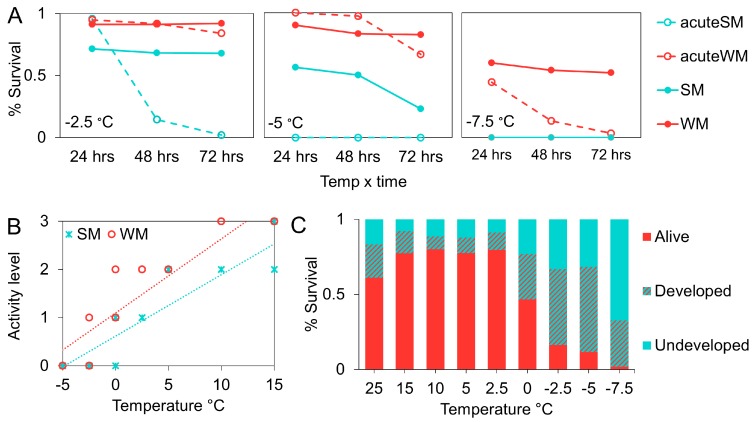
Mean percent survival at sub-freezing temperatures was compared among flies undergoing dynamic acclimation (SM and WM) with those acutely exposed (acute SM and acute WM) (**A**). The relationship between temperature and mean activity level in adult *D. suzukii* (**B**). Greater values indicate greater activity level. The dashed lines indicate best fit for SM (blue line) and WM (red line) flies. The effect of gradual cooling on pupal survival and development (**C**).

Below 0 °C, we included two extra control groups to directly compare survival in our SM and WM groups with flies in an acute exposure scenario. Flies in the “acute SM” group were moved from the 25 °C colony chamber to the cold chamber directly, while the “acute WM” group was moved from the 15 °C chamber. The acute SM (z = 10.04, *p* < 0.001; Figure 7A) and acute WM flies (z =4.3, *p* = 0.0001; Figure 7A) showed significantly greater mortality relative to the dynamic acclimation treatments. However, the acute WM flies survived in higher proportions than the SM flies following dynamic acclimation (z = −9.4, *p* < 0.001).

There was a positive relationship between activity level and temperature (F = 215, df = 5, 94, *p* < 0.001; Figure 7B). We observed complete chill coma below 0 °C in SM flies and −2.5 °C in WM flies. Flies in the thermal control treatments (C-SM and C-WM flies) displayed constant activity levels throughout the course of the experiment, indicating that time/age did not affect activity level. C-WM flies were consistently more active at 15 °C than C-SM flies (t = 4.25, *p* < 0.001). 

Survival among pupae was temperature dependent (χ^2^ = 211.7, df = 1,7, *p* < 0.001; Figure 7C). Near freezing, from 2.5 °C to 0 °C, there was a significant decline in survival from approximately 80% to 47% (χ^2^ = 42.56, df = 1, *p* < 0.001). There was another sharp decrease in survival when the temperature was reduced to −2.5 °C (χ^2^ = 38.75, df = 1, *p* < 0.001). Between −2.5 °C and −5 °C survival was similar, around 15% (χ^2^ = 1.51, df = 1, *p* = 0.23). We observed 1% survival at the lowest temperature we tested, −7.5 °C (χ^2^ = 8.89, df = 1, *p* = 0.003).

## 4. Discussion

The physiological degree of cold hardening in *D. suzukii* is likely dependent on the acclimation temperature to which the insects are exposed during larval and early adult development. The current study investigated the comparative survival of *D. suzukii* after static and dynamic acclimation protocols in adult and pupal flies and demonstrated that gradual reductions in temperature were associated with greater survival outcomes among SM and WM flies, than static acclimation protocols in which a single temperature was employed. Our study suggests that the thermal limits of survival in *D. suzukii* ought not to be defined by one value. Rather, thermal susceptibility likely varies with lifestage and experience, and as such, there may be variable LT^50^ values for this species depending on those factors. Cold hardening and the capacity for overwintering survival may be profoundly affected by how rapidly cooling occurs, and gradual cooling may increase cold tolerance in this species. More research is needed to determine the role of fluctuating daily temperatures in the physiogenesis of cold tolerance in *D. suzukii*, as it is unclear what effect thermal instability may have on survival outcomes. It also unclear whether thermal susceptibility is similarly affected by gradual warming, thereby extending the limits of survival in warmer climates.

In the first two experiments, we compared survival among acclimated and non-acclimated flies using a static acclimation protocol. Developmentally acclimated WM flies survived the best at cold temperatures, followed by cold-acclimated SM flies. Non-acclimated SM flies did not survive prolonged exposure to temperatures below 4.4 °C. Below freezing, all non-acclimated flies died within 24 h. These data are consistent with previous reports of varying levels of cold hardiness in adult *D. suzukii* [20,22,29,32,33,67]. At least some of the physiological changes associated with short-term adult cold acclimation appear to occur rapidly in Drosophilids. In *D. melanogaster*, short-term adult acclimation is sufficient to induce a 22% increase in cold tolerance [38]. We found similar effects in *D. suzukii*, where short-term acclimation was sufficient to induce moderate protection. Cold-acclimated SM flies survived longer than non-acclimated flies above −5 °C. However, WM flies, which developed under cool conditions, were much more robust and survived the longest.

We observed similar results when we manipulated cold hardiness in pupal *D. suzukii*. Acquired cold hardiness improved the survival of pupal *D. suzukii* after chronic exposure to cold temperatures. Acclimated pupae survived up to a week at just above and below freezing, indicating greater cold tolerance than was previously identified [22,31,37,45]. This effect was most pronounced after 24 h exposure when the survival of acclimated pupae became greater than survival among non-acclimated insects. Greater initial survival among non-acclimated flies in our control groups, which were allowed to eclose without cold exposure, indicates that acclimation may be a stressor that results in death among some developing flies. This is consistent with previous reports of high juvenile mortality at low temperatures [45]. However, we observed that among the pupae that failed to eclose, those that were previously acclimated showed more advanced development than non-acclimated flies. In non-acclimated flies, pupal development appeared to arrest below 4.4 °C, whereas acclimated pupae showed nearly complete development, with visible body structures such as wings, legs, and eyes at temperatures as low as −3.5 °C. Although we did not directly compare juvenile and adult cold susceptibilities, we did note the comparative survival among acclimated and non-acclimated insects at these two life stages. Juveniles were susceptible to freezing temperatures below −5 °C, but so were adult flies in their respective assays. Both groups showed consistent survival above −5 °C and the patterns for survival were more similar than we expected. This is surprising given that juvenile susceptibility is common among drosophilids [43]. While our data suggest that pupae are more cold tolerant than previously observed, extended periods at or below freezing appear fatal, indicating that juvenile overwintering diapause is unlikely.

In the final experiment, we re-assessed survival following dynamic acclimation, or a slow progression toward freezing temperatures. We compared SM and WM fly survival daily and found improved survival outcomes among both groups. While WM survival was greater than SM survival throughout the study, there was not a significant decline in survival among SM flies until the temperature was reduced to −5 °C. In contrast, our initial tests using static acclimation suggested SM flies were susceptible at −1.87 °C. Above −5 °C, survival followed a pattern of gradual and mild decline consistent with our control flies, which were held at 15 °C for the duration of the study. Among those flies too, WM survival was greater than SM survival. The thermal limit for WM flies was lower than SM flies, and did not start to decline until temperatures were reduced even further, to −7.5 °C. While all flies in the SM treatment were dead within 1 d at −7.5 °C, approximately 50% of our WM flies were still alive after 3 days. This is a dramatic increase in survival compared to our initial thermal susceptibility assessments, which suggested a lower lethal limit of −6.7 °C. Similarly, pupal survival improved following gradual cooling. Although survival at temperatures below freezing was low, we did observe some level of survival at all of our tested temperatures, indicating greater cold tolerance than previously demonstrated.

Gradual cooling is likely a better reflection of the cold hardening *D. suzukii* would experience in nature [50], and appears to confer greater protection against low temperatures than static acclimation [68,69]. The internal physiogenesis that occurs during cold hardening is likely an ongoing, dynamic process proportional to the degree of cold to which the insects are exposed [55]. As temperatures decrease in the late fall and early winter, local populations of *D. suzukii* may become increasingly adapted to the cool weather as physiological changes, such as increases in carbohydrate stores develop [30,32,41]. In *D. melanogaster*, gradual acclimation, like developmental acclimation, appears to involve long-lasting physiological changes in gene expression, thereby promoting cold hardiness [41,68]. Indeed, our data suggest that SM flies may be moderately cold hardened without displaying the external phenotypic traits associated with WM flies. In this species we should perhaps distinguish between the morphogenesis associated with external WM traits and the physiogenesis that may occur in both WM and SM flies [55].

In addition to physiological adaptations via variation in phenotypic expression, it is likely that *D. suzukii* uses behavioral and environmental strategies to thermo-modulate. The Japanese Drosophilid, *D. curviceps,* is known to thermo-modulate via seasonal migration between the warmer lowlands and the cooler highlands [70]. Similar migration could occur in *D. suzukii* allowing it to avoid the most extreme low temperatures in a given area. Indeed, there is evidence that *D. suzukii* moves between crop land and adjacent wooded areas during peak field season [71,72,73,74,75]. Similarly, there is reason to believe that these insects may overwinter in habitats such as leaf litter [20,25,76], which provides thermal insulation and keeps ground temperatures within a few degrees of freezing throughout the winter [59,77,78]. In the bertha armyworm, *Mamestra configurata* (Lepidoptera: Noctuidae), and the rice water weevil, *Lissorhoptrus oryzophilus* (Coleoptera: Curculionidae), survival of overwintering immatures is greatest in areas of deep snow pack [79,80]. Similarly, *D. suzukii* may access insulation in the top soil layers, during periods of heavy snow and extreme cold, such that the temperature is relatively stable at 0 °C. This freeze-avoidant strategy may allow WM *D. suzukii* to successfully overwinter by avoiding incompatibly low temperatures. 

## 5. Conclusions

Given the economic impact *D. suzukii* has had in temperate growing regions, these data may have important implications for offseason population monitoring and management. Future research ought to identify overwintering hot-spots that occur near agricultural zones, track migration during late fall and early spring, and look for novel methods of population suppression that exploit the ecological niche this insect inhabits. There are concerns that *D. suzukii* may utilize urban refugia [11,14,20,21,22,81], which may provide access to heat and food and therefore serve as a population reservoir. These environments should be studied closely to determine if there is an opportunity to reduce overwintering success in this invasive, economically destructive species.

Increased globalization has resulted in the frequent invasion of non-native pest species, such as *D. suzukii* into foreign environments. In addition, climate change is associated with marked range expansion of pests into previously uninhabitable climates closer to the poles [59,63]. While predictive models [4,63] have suggested that *D. suzukii* movement may be limited by narrow thermal thresholds between approximately 10–30 °C [23,82], our data suggests that such models may benefit from taking dynamic acclimation, and the plasiticity of the cold hardening response in *D. suzukii*, into account. The ability to adaptively cope with thermal extremes may be a previously unrecognized risk factor for expanding latitudinal invasion [63] and new continental invasion events by *D. suzukii* [4].

## Figures and Tables

**Table 1:** Sample sizes and replicate structure for each adult static acclimation treatment group.

Acclimation	Phenotype	Total No. Insects Tested per Treatment N = (#Replicates)					
		4.4 °C	1.7 °C	−1.1 °C	−3.9 °C	−6.7 °C	−9.4 °C
Yes	WM	78 (3)	71 (3)	69 (3)	75 (3)	72 (3)	75 (3)
Yes	SM	131 (5)	142 (5)	130 (5)	138 (5)	124 (5)	125 (5)
No	SM	129 (5)	127 (5)	127 (5)	135 (5)	108 (5)	125 (5)
